# The Role of Multiparametric MRI Radiomics for Preoperative Prediction of Axillary Lymph Node Metastasis in Patients With Invasive Breast Cancer: A Comparative Study

**DOI:** 10.1002/cai2.70022

**Published:** 2025-07-13

**Authors:** Qingcong Kong, Yongxin Chen, Yi Sui, Siyi Chen, Xinghan Lv, Wenjie Tang, Zhidan Zhong, Xiaomeng Yu, Kuiming Jiang, Lei Zhang, Jianning Chen, Jie Qin, Yuan Guo

**Affiliations:** ^1^ Department of Radiology, The Third Affiliated Hospital Sun Yat‐Sen University Guangzhou Guangdong China; ^2^ Department of Radiology, Guangzhou First People's Hospital South China University of Technology Guangzhou Guangdong China; ^3^ Department of Radiology Guangzhou Women and Children's Medical Center Guangzhou Guangdong China; ^4^ Department of Ultrasound Guangdong Women and Children Hospital Guangzhou Guangdong China; ^5^ Department of Radiology Guangdong Women and Children Hospital Guangzhou Guangdong China; ^6^ Department of Diagnostic Radiology and Nuclear Medicine University of Maryland School of Medicine Baltimore Maryland USA; ^7^ Department of Pathology, The Third Affiliated Hospital Sun Yat‐Sen University Guangzhou Guangdong China

**Keywords:** axillary lymph nodes, invasive breast cancer, multiparametric magnetic resonance imaging, nomogram, radiomics

## Abstract

**Background:**

The predictive value of different MRI sequences for axillary lymph node metastasis (ALNM) in patients with invasive breast cancer remains unclear. This study compared the performance of radiomics models based on individual and combined MRI sequences for the preoperative prediction of ALNM and evaluated the clinical application value of the optimal model.

**Methods:**

This retrospective study included 454 patients (mean ± SD age 50.9 ± 10.7 years) diagnosed with invasive breast cancer from two centers, with 382 patients from Center 1 (training cohort) and 72 patients from Center 2 (external test cohort). Tumor segmentation and radiomics feature extraction were performed on T2‐weighted imaging (T2WI), diffusion‐weighted imaging (DWI), and dynamic contrast‐enhanced (DCE) images. The least absolute shrinkage and selection operator with 10‐fold cross‐validation was used for feature selection and radiomics score construction. Three single‐sequence models and one multi‐sequence radiomics model were developed, and the optimal model was combined with conventional MRI features to create a combined MRI model. The combined model's performance was compared to radiologists' diagnoses. A nomogram was developed based on the optimal model and correlated with prognosis using the Kaplan–Meier curve and Cox proportional hazard regression. Model performance was evaluated using area under the curve (AUC); DeLong's test was used for comparison.

**Results:**

In the external test cohort, the DCE model showed the highest performance (AUC = 0.76) but was not significantly different from T2WI (AUC = 0.72) and DWI (AUC = 0.70) (all *p* > 0.05). The combined radiomics model achieved an AUC of 0.82, outperforming DWI and T2WI (*p* < 0.05), but was not significantly different from the DCE model (*p* > 0.05). The combined MRI model demonstrated the highest AUC of 0.84 and notably improved radiologist diagnostic accuracy. A nomogram based on the combined MRI model was developed to assist clinical decision‐making by providing individualized risk predictions. The higher‐risk group based on the model's predictive probability showed a significantly worse prognosis (*p* < 0.001).

**Conclusion:**

The combined radiomics model outperformed single‐sequence models in predicting ALNM. The combined MRI model demonstrated the highest performance, improving diagnostic accuracy and showing potential for prognostic prediction.

## Graphical abstract

This study compared the preoperative noninvasive prediction of axillary lymph node metastasis in patients with invasive breast cancer using radiomics derived from single and multi‐sequence MRI. The combined radiomics model outperformed the single‐sequence models, with the DCE model performing better among the single‐sequence models. The combined MRI model achieved the highest performance, improving diagnostic accuracy and showing potential for prognostic prediction.

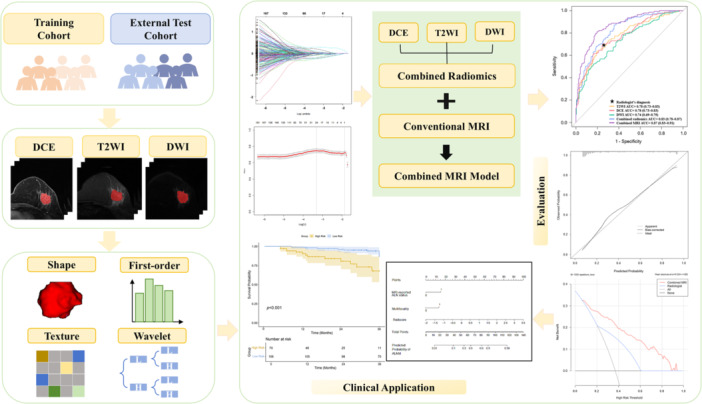

**Abbreviations**
ALN: axillary lymph nodes
ALND: axillary lymph node dissection
ALNM: axillary lymph node metastasis
AUC: area under the curve
DCA: decision curve analysis
DCE: dynamic contrast‐enhancement
DFS: disease‐free survival
DWI: diffusion‐weighted imaging
ER: estrogen receptor
HER2: human epidermal growth factor receptor‐2
ICC: interobserver correlation coefficient
IHC: immunohistochemistry
KM: Kaplan–Meier
MRI: magnetic resonance imaging
pALN: pathologic axillary lymph node
PR: progesterone receptor
ROC curve: receiver operating characteristic curve
ROI: region of interest
SLNB: sentinel lymph node biopsy
T2WI: T2‐weighted imaging

## 1 Introduction

Breast cancer is the most commonly diagnosed cancer among women globally [1]. The presence of metastasis in axillary lymph nodes (ALN) in breast cancer patients is associated with higher recurrence rates and increased mortality [2]. Accurate evaluation of ALN status is crucial for determining the stage of the disease, formulating treatment plans, and predicting outcomes for patients [3]. Sentinel lymph node biopsy (SLNB) is the standard for assessing ALN involvement and aids clinicians in determining the necessity of ALN dissection (ALND) and planning further therapeutic approaches [4]. However, approximately 43%–65% of patients with positive sentinel lymph nodes undergo additional axillary surgeries that may not be needed, extending surgical duration and adding to patient distress [5]. Both SLNB and ALND are invasive procedures that can lead to complications such as lymphedema and pain [6, 7]. This underscores the critical need for noninvasive techniques for the preoperative assessment of ALN status, which could help tailor treatments more precisely and reduce unnecessary surgical interventions.

The development of imaging modalities is rapidly advancing to address the need for preoperative, noninvasive ALN status prediction. Imaging techniques such as mammography, ultrasound, and positron emission tomography/computed tomography are frequently used; however, their use in detecting ALN metastasis (ALNM) is limited by high false‐negative rates and suboptimal sensitivity [8, 9, 10]. Magnetic resonance imaging (MRI), a nonradiographic technique, offers enhanced soft‐tissue contrast and superior sensitivity, showing significant potential in accurately evaluating ALNM [3]. However, the traditional reliance on visual analysis of morphological changes on MRI may overlook vital biological characteristics. Radiomics, which extracts high‐throughput quantitative data from medical images, has identified valuable noninvasive biomarkers for clinical decision‐making [11, 12]. Recent studies demonstrated the effectiveness of MRI‐based radiomics in accurately diagnosing breast lesions, distinguishing molecular subtypes, and predicting ALNM with notable precision [13, 14, 15].

Radiomics based on dynamic contrast‐enhancement (DCE) imaging has shown promise in predicting ALNM status in breast cancer, with area under the curve (AUC) values ranging from 0.74 to 0.81 in validation cohorts [16, 17]. A previous study using multiparametric MRI radiomics indicated that diffusion‐weighted imaging (DWI) performed similarly to DCE and was superior to T2‐weighted imaging (T2WI) [18]. However, models integrating multiple sequences did not demonstrate significant improvement over single‐sequence models. This finding contradicts the results reported in other studies [19]. As a result, the relative efficacy of different MRI sequences for predicting ALNM remains unclear, and whether models integrating multiple sequences show improved predictive performance over single‐sequence models is still a topic of debate.

The objective of this study was to compare the predictive performances of radiomics models based on different MRI sequences and assess the potential added value of multi‐sequence models. Additionally, we integrated the optimal radiomics model with conventional MRI features to further enhance predictive performance and improve clinical applicability by exploring its association with prognosis.

## 2 Methods

### 2.1 Study Sample

**Figure 1:**
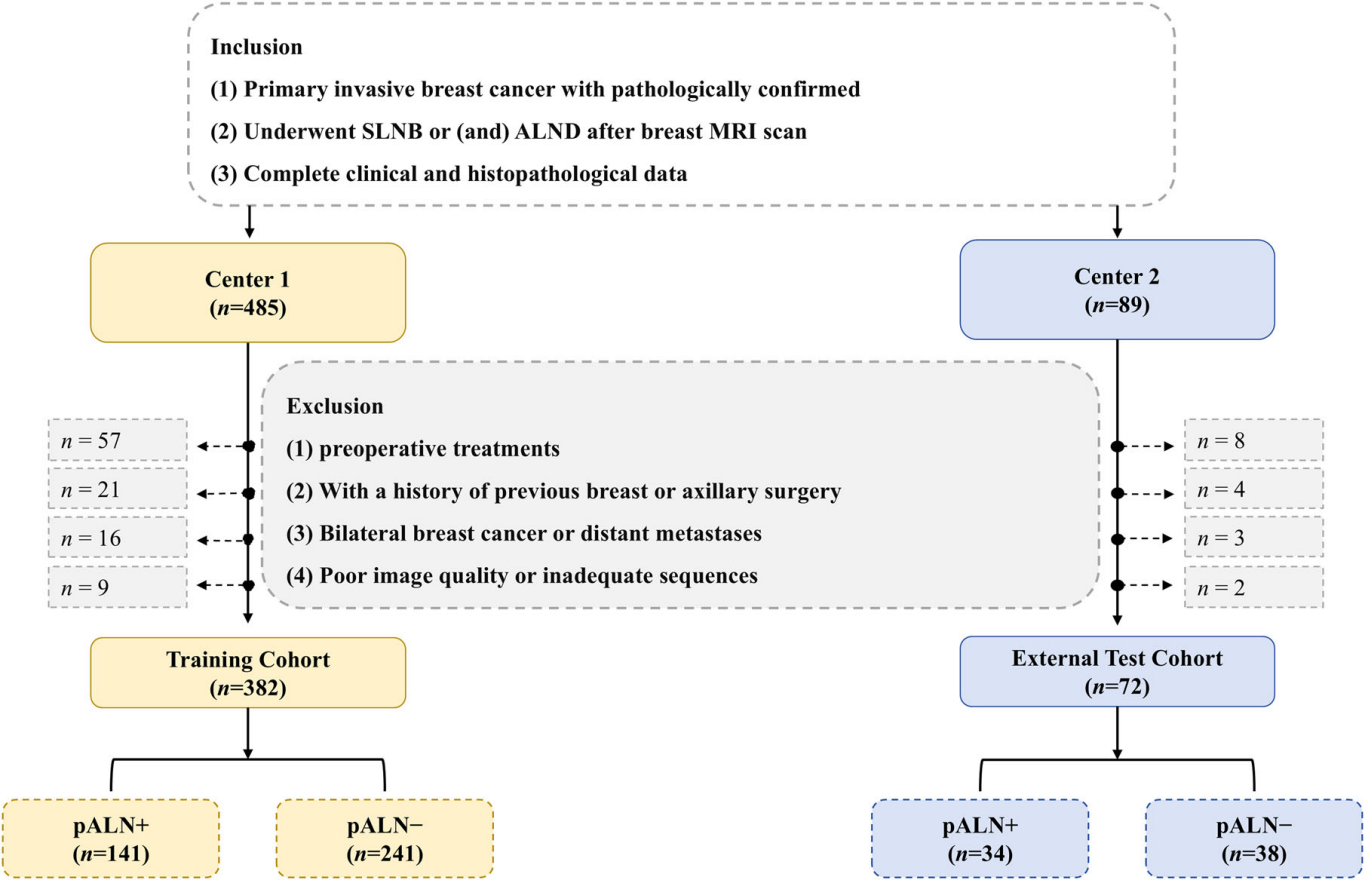
Flowchart of the patient inclusion and exclusion criteria. ALND, axillary lymph node dissection; MRI, magnetic resonance imaging; pALN+, pathologic axillary lymph node positive; pALN−, pathologic axillary lymph node negative; SLNB, sentinel lymph node biopsy.

**Figure 2:**
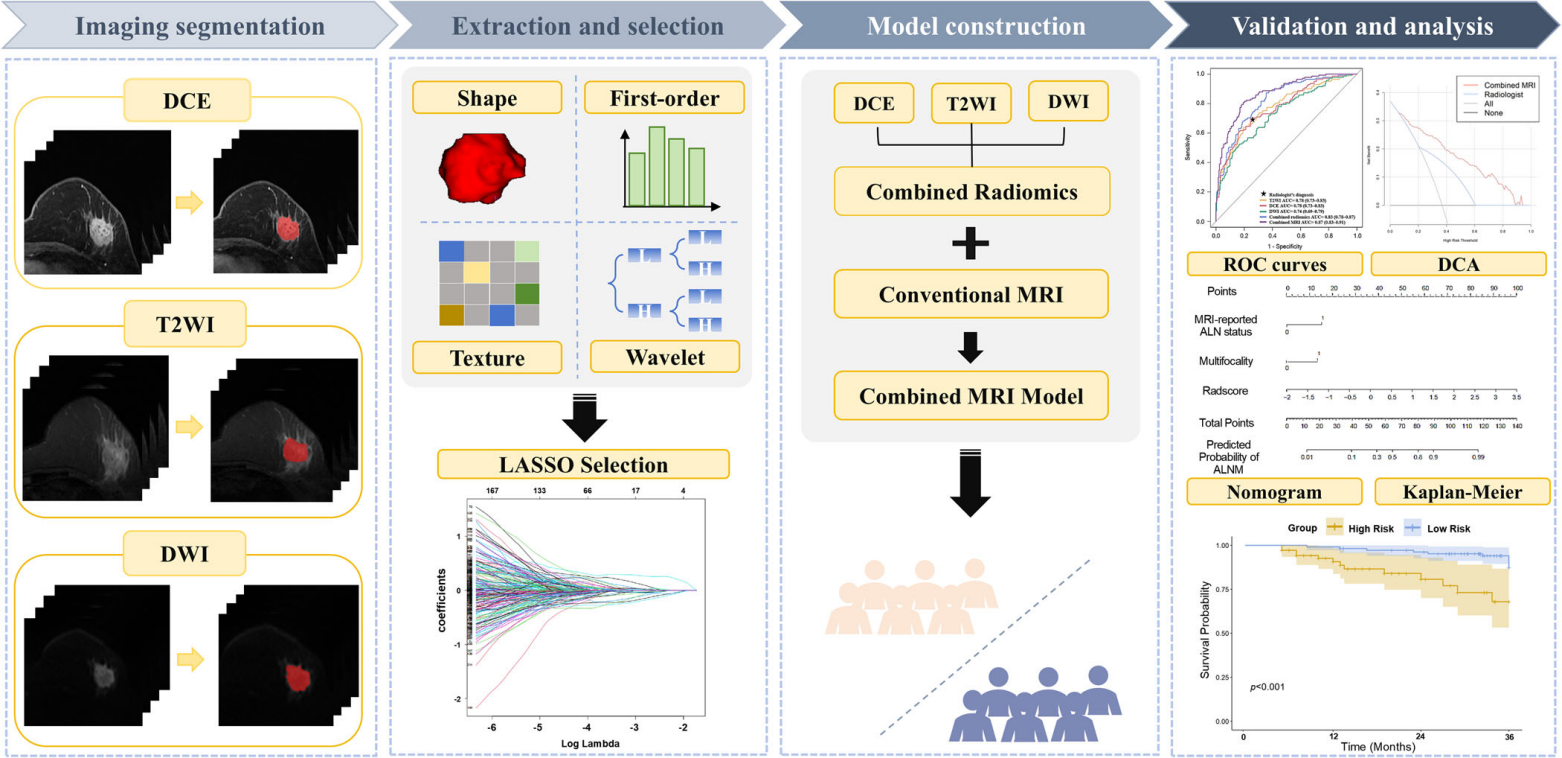
The workflow of this study. This study included imaging segmentation, radiomics features extraction and selection, model construction, and validation and analysis. DCA, decision curve analysis; DCE, dynamic contrast‐enhancement; DWI, diffusion‐weighted imaging; T2WI, T2‐weighted imaging.

The patient recruitment process is shown in Figure 1. Patients with invasive breast cancer were recruited from two centers. Both centers used electronic medical records to identify patients diagnosed with invasive breast cancer who had undergone ALN assessment through SLNB and/or ALND and who received multiparametric breast MRI (from November 2018 to November 2023 at Center 1 and from November 2022 to November 2023 at Center 2). This search identified 485 patients at Center 1 and 89 at Center 2. The exclusion criteria were as follows: (1) breast‐related treatment like radiotherapy or neoadjuvant therapy before MRI scan (Center 1, *n* = 57; Center 2, *n* = 8); (2) prior breast or axillary surgery (Center 1, *n* = 21; Center 2, *n* = 4); (3) bilateral breast cancer or distant metastasis (Center 1, *n* = 16; Center 2, *n* = 3); and (4) poor image quality or incomplete imaging sequences (Center 1, *n* = 9; Center 2, *n* = 2). Following these exclusions, a total of 454 patients were enrolled in the study, including 382 from Center 1 (the training cohort) and 72 from Center 2 (the independent external test cohort). The study workflow is detailed in Figure 2.

### 2.2 Clinicopathologic Data

Clinicopathologic data were sourced from medical records, with the pathologic ALN (pALN) status determined through SLNB and/or ALND. pALN positivity was classified on the basis of the presence of micrometastasis (0.2–2 mm) or macrometastasis (> 2 mm) [17, 20].

Additional clinicopathologic parameters included patient age, menstrual status, and biomarker expressions such as human epidermal growth factor receptor‐2 (HER2), estrogen receptor (ER), progesterone receptor (PR), and Ki‐67, assessed from surgical specimens. ER or PR positivity was identified by more than 1% of nuclei showing positive staining via immunohistochemistry (IHC) [21]. HER2 was considered positive with an IHC score of 3+ or if fluorescence in situ hybridization showed an amplification ratio ≥ 2.0 [22]. Ki‐67 expression was categorized as high or low with a threshold of 14% [17].

### 2.3 MRI Examination

MRI examinations were conducted using two different 1.5 T MRI systems equipped with dedicated bilateral breast coils. Patients were imaged in the prone position. The MRI protocols included fat‐suppressed T2WI, DCE‐MRI, and DWI with b‐values of 0 and 800 s/mm^2^. For DCE‐MRI, Center 1 used a gadolinium‐based contrast agent (Gd‐DTPA, Magnevist; Bayer HealthCare, Germany) administered at a rate of 1.5 mL/s and a dosage of 0.2 mL/kg of body weight, followed by a 20‐mL saline flush using a high‐pressure syringe. Center 2 administered the same agent at a dosage of 0.2 mmol/kg body weight with an infusion rate of 2 mL/s, followed by a 15‐mL saline flush. Acquisition parameters for these procedures are detailed in Supporting Information: Table S1.

### 2.4 Conventional MRI Features Assessment

Two radiologists (C.Y.X. and S.Y., both with 3 years of posttraining experience), blinded to pALN status, independently reviewed the MRI results from both centers to evaluate conventional features. Conventional features included tumor size, laterality, amount of fibroglandular tissue, background parenchymal enhancement, multifocality, tumor shape, tumor margins, internal enhancement, peritumoral edema on T2WI, and MRI‐reported ALN status.

Tumor size was quantified by measuring the lesion's largest diameter during the early phase of DCE‐MRI. Peritumoral edema was identified by a hyperintense signal adjacent to the tumor on axial or sagittal T2‐weighted images. MRI‐reported ALN was considered abnormal if any of the following criteria was satisfied [3, 23, 24]: (1) loss of the fatty hilum; (2) ALN short‐axis diameter greater than 10 mm; (3) a longest‐to‐shortest axis ratio less than 2; (4) asymmetry relative to the contralateral side; (5) eccentric cortical thickening, and (6) irregular margins. These criteria also guided radiologists in diagnosing ALN status. All other features were evaluated following the Breast Imaging Reporting and Data System Atlas, 5th edition. For cases with multifocal lesions, only the largest lesion was considered for image analysis.

In cases of disagreement on categorical assessments, the two primary radiologists attempted to reach a consensus. If unresolved, a third, more experienced radiologist (K.Q.C. with 17 years of post‐training experience) made the final decision. Tumor size measurements from both radiologists were averaged to establish a final size for each tumor.

### 2.5 Tumor Segmentation

A breast MRI radiologist (C.Y.X. with 3 years of post‐training experience) independently segmented the entire tumor region of interest (ROI) using ITK‐SNAP software (version 4.0.1) across DCE, T2WI, and DWI sequences. For DCE‐MRI, ROIs were manually delineated on axial slices during the early phase of DCE (approximately 90 s after contrast injection) to maximize contrast differentiation between the tumor and surrounding tissues [25, 26]. These delineations were used as references to segment ROIs on T2WI and DWI (b‐value of 800 s/mm^2^). The segmentation excluded edema, blood vessels, and normal fibroglandular tissue, focusing solely on the tumor's entirety. In cases of multiple lesions, only the largest was segmented. To assess interobserver agreement, a second radiologist (G.Y. with 17 years of post‐training experience) independently segmented ROIs for a random subset of 50 patients from the training cohort. The consistency of radiomics features between the two radiologists was quantified using the interobserver correlation coefficient (ICC).

### 2.6 Extraction and Selection of Radiomics Features

For each tumor, we extracted 3390 radiomics features from the ROIs placed on T2WI, DWI, and second‐phase DCE (each contributing 1130 features) using the PyRadiomics package. Before extracting these features, all signal intensities within the ROIs underwent z‐score normalization to standardize the data. Initially, only features with ICCs ≥ 0.75, indicating good‐to‐excellent agreement between radiologists, were retained [27]. Features with a correlation coefficient greater than 0.9 were then eliminated through Pearson correlation analysis. The least absolute shrinkage and selection operator with 10‐fold cross‐validation was used to eliminate features with zero coefficients and identify the most significant features for the model.

### 2.7 Construction of Models

The radiomics models generated a radiomics score (hereafter, radscore), calculated by combining the selected radiomics features, which were weighted using the coefficients obtained from the least absolute shrinkage and selection operator regression. Individual radiomics models were developed for each imaging sequence—DCE, T2WI, and DWI—and a combined model that integrated all three sequences (T2WI+DCE+DWI) was also created.

A combined MRI model was constructed by incorporating the most effective radiomics model with significant conventional MRI features. These features were identified through univariate and multivariate logistic regression analyses; variables exhibiting a *p*‐value < 0.05 were included. The predictive model with the highest performance was used to develop a nomogram, providing a visual representation of the prediction outcomes.

### 2.8 Survival Analysis

To evaluate the clinical applicability of the best predictive model, we investigated the association between disease‐free survival (DFS) and the model's risk scores. DFS was defined as the time interval between the date of surgery and the occurrence of tumor recurrence, distant metastasis, or death [28]. Patient follow‐up and recurrence data were obtained from electronic medical records, with the last follow‐up date being June 2024. Only patients with at least 3 years between the surgery date and the last follow‐up date were included in the analysis. Patients who did not experience recurrence or were lost to follow‐up during this period were considered as censored observations.

The predictive probabilities from the optimal model were used to calculate individual risk scores. These risk scores were categorized into low‐risk and high‐risk groups, using a cutoff value determined by the Youden Index. Kaplan–Meier (KM) curves were generated to assess the prognostic differences between the two risk groups.

### 2.9 Statistical Analysis

All statistical analyses were conducted using SPSS (version 26.0) and R software (version 4.1.2). Differences between groups were evaluated by comparing continuous variables with the Student's *t*‐test or Mann–Whitney *U* test and categorical variables with the chi‐square test or Fisher's exact test. Interreader agreement for tumor size was assessed across two centers using the ICC [27], while agreement for other conventional MRI features was evaluated using kappa coefficients [29]. The effectiveness of the models was evaluated using the receiver operating characteristic (ROC) curve and AUC, and the DeLong test was used to compare performances across different models. Calibration curves were generated to evaluate the concordance between the predicted probabilities and actual outcomes of the models. The clinical utility of the nomogram was determined through decision curve analysis (DCA). The log‐rank test was used to compare the differences in survival between the risk groups based on the KM curve. Cox proportional hazards regression analysis was applied to assess the relationship between risk scores and prognosis. Statistical significance was set at a two‐tailed *p* < 0.05.

## 3 Results

### 3.1 Patient Characteristics

This study included 454 patients diagnosed with invasive breast cancer from two centers. Among the 382 patients from Center 1 (the training cohort), 141 (36.9%) tested positive for pALN metastasis; among the 72 patients from Center 2 (the external test cohort), 34 (47.2%) exhibited pALN metastasis positivity. There were no statistically significant differences in the clinicopathological characteristics between the patient groups (*p* > 0.05) (Supporting Information: Table S2).

The interreader consistency for conventional MRI features is shown in Supporting Information: Table S3. The agreement for tumor size was excellent (ICC = 0.94) and for categorical features was substantial to almost perfect (kappa, 0.78–0.91).

Significant differences were observed between patients with positive and negative pALN concerning tumor size, multifocality, peritumoral edema on T2WI, and the MRI‐reported status of ALN in both the training and external test cohorts (*p* < 0.05) (Table 1).

**Table 1:** The conventional MRI features of patients in the two cohorts.

	Training cohort (*n* = 382)			External test cohort (*n* = 72)		
Characteristics	pALN+(*n* = 141)	pALN−(*n* = 241)	*p*‐value	pALN+(*n* = 34)	pALN−(*n* = 38)	*p*‐value
Tumor size (mm) (mean ± SD)	32.3 ± 16.2	21.5 ± 8.7	**< 0.001**	33.4 ± 15.3	22.9 ± 10.6	**0.001**
Location, *n* (%)			0.47			0.76
Left	75 (53.2)	119 (49.4)		14 (41.2)	17 (44.7)	
Right	66 (46.8)	122 (50.6)		20 (58.8)	21 (55.3)	
FGT, *n* (%)			0.48			0.17
Fatty/scattered	72 (51.1)	114 (47.3)		11 (32.4)	7 (18.4)	
Heterogeneous/extremely dense	69 (48.9)	127 (52.7)		23 (67.6)	31 (81.6)	
BPE, *n* (%)			0.17			0.59*
Minimal or mild	117 (83.0)	212 (88.0)		31 (91.2)	32 (84.2)	
Moderate or marked	24 (17.0)	29 (12.0)		3 (8.8)	6 (15.8)	
Multifocality			**< 0.001**			**0.02**
Multiple	55 (39.0)	29 (12.0)		15 (44.1)	7 (18.4)	
Single	86 (61.0)	212 (88.0)		19 (55.9)	31 (81.6)	
Tumor shape			0.15			0.07
Round to oval	26 (18.4)	60 (24.9)		4 (11.8)	11 (28.9)	
Irregular	115 (81.6)	181 (75.1)		30 (88.2)	27 (71.1)	
Tumor margin			0.07			0.34*
Circumscribed	14 (9.9)	40 (16.6)		2 (5.9)	6 (15.8)	
Not circumscribed	127 (90.1)	201 (83.4)		32 (94.1)	32 (84.2)	
Mass internal enhancement			0.05			0.78*
Homo‐ or heterogeneous	136 (96.5)	220 (91.3)		32 (94.1)	34 (89.5)	
Rim	5 (3.5)	21 (8.7)		2 (5.9)	4 (10.5)	
Peritumoral edema on T2WI			**0.03**			**0.02**
Present	74 (52.5)	99 (41.1)		22 (64.7)	14 (36.8)	
Absent	67 (47.5)	142 (58.9)		12 (35.3)	24 (63.2)	
MRI‐reported ALN status			**< 0.001**			**0.001**
Abnormal	95 (67.4)	62 (25.7)		26 (76.5)	14 (36.8)	
Normal	46 (32.6)	179 (74.3)		8 (23.5)	24 (63.2)	

Abbreviations: ALN, axillary lymph nodes; MRI, magnetic resonance imaging; pALN+, pathologic axillary lymph node positive; pALN−, pathologic axillary lymph node negative; T2WI, T2‐weighted imaging.

* *p* values were calculated by Fisher's exact test.

### 3.2 Construction of Radiomics Models and Evaluation

A selection of radiomics features was finalized for the construction of single‐sequence models: 12 from DCE, 15 from T2WI, and 3 from DWI (Supporting Information: Table S4). For the single‐sequence radiomics models, the AUCs for T2WI, DWI, and DCE were 0.78 (95% confidence interval [CI]: 0.73–0.83), 0.74 (95% CI: 0.69–0.79), and 0.78 (95% CI: 0.73–0.83) in the training cohort and 0.72 (95% CI: 0.60–0.84), 0.70 (95% CI: 0.58–0.82), and 0.76 (95% CI: 0.65–0.87) in the external test cohort, respectively (Table 2). In the training cohort, DCE demonstrated significantly better performance than DWI (*p* < 0.05) and was comparable to T2WI (*p* > 0.05). In the external test cohort, although DCE showed the highest performance, the differences compared with T2WI and DWI were not statistically significant (*p* > 0.05).

**Table 2:** The performance of different radiomics models for predicting ALNM in the two cohorts.

Model	AUC (95% CI)	SEN	SPE	ACC	*p*‐value^a^	*p*‐value^b^
Training cohort						
T2WI	0.78 (0.73–0.83)	0.64	0.81	0.74	0.04^c^	0.001
DWI	0.74 (0.69–0.79)	0.53	0.83	0.72	0.03^d^	< 0.001
DCE	0.78 (0.73–0.83)	0.70	0.75	0.73	0.98^e^	< 0.001
Combined radiomics	0.83 (0.78–0.87)	0.87	0.64	0.73	—	—
External test cohort						
T2WI	0.72 (0.60–0.84)	0.47	0.90	0.69	0.67^c^	0.02
DWI	0.70 (0.58–0.82)	0.71	0.66	0.68	0.21^d^	0.01
DCE	0.76 (0.65–0.87)	0.56	0.87	0.72	0.42^e^	0.23
Combined radiomics	0.82 (0.72–0.92)	0.85	0.74	0.79	—	—

*Note: p*‐value calculated using the DeLong test.

Abbreviations: ACC, accuracy; AUC, area under curve; CI, confidence interval; DCE, dynamic contrast‐enhancement; DWI, diffusion‐weighted imaging; SEN, sensitivity; SPE, specificity; T2WI, T2‐weighted imaging.

^a^ Comparison of AUC between each single‐sequence radiomics model.

^b^ Comparison of AUC between the single‐sequence radiomics model and combined radiomics model.

^c^ Comparison of AUC between the T2WI radiomics model and DWI radiomics model.

^d^ Comparison of AUC between the DWI radiomics model and DCE radiomics model.

^e^ Comparison of AUC between the DCE radiomics model and T2WI radiomics model.

The combined radiomics model, integrating 24 features—9 from DCE, 5 from DWI, and 10 from T2WI (Supporting Information: Table S5)—achieved an AUC of 0.83 (95% CI: 0.78–0.87) in the training cohort. This performance significantly surpassed that of the single‐sequence models (all *p* < 0.05). In the external test cohort, the model reached an AUC of 0.82 (95% CI: 0.72–0.92), which was significantly better than those achieved by T2WI and DWI (all *p* < 0.05) and on par with DCE (*p* > 0.05) (Table 2).

### 3.3 Construction of the Combined MRI Model and Evaluation

In univariate logistic regression, larger tumor size (OR = 1.08, 95% CI: 1.06–1.11), multifocality (OR = 4.68, 95% CI: 2.79–7.82), presence of peritumoral edema (OR = 1.58, 95% CI: 1.04–2.41), MRI‐reported abnormal ALN (OR = 5.96, 95% CI: 3.78–9.40), and higher radscore (OR = 5.49, 95% CI: 3.79–7.96) were significantly associated with increased pALN metastasis positivity (*p* < 0.05).

**Figure 3:**
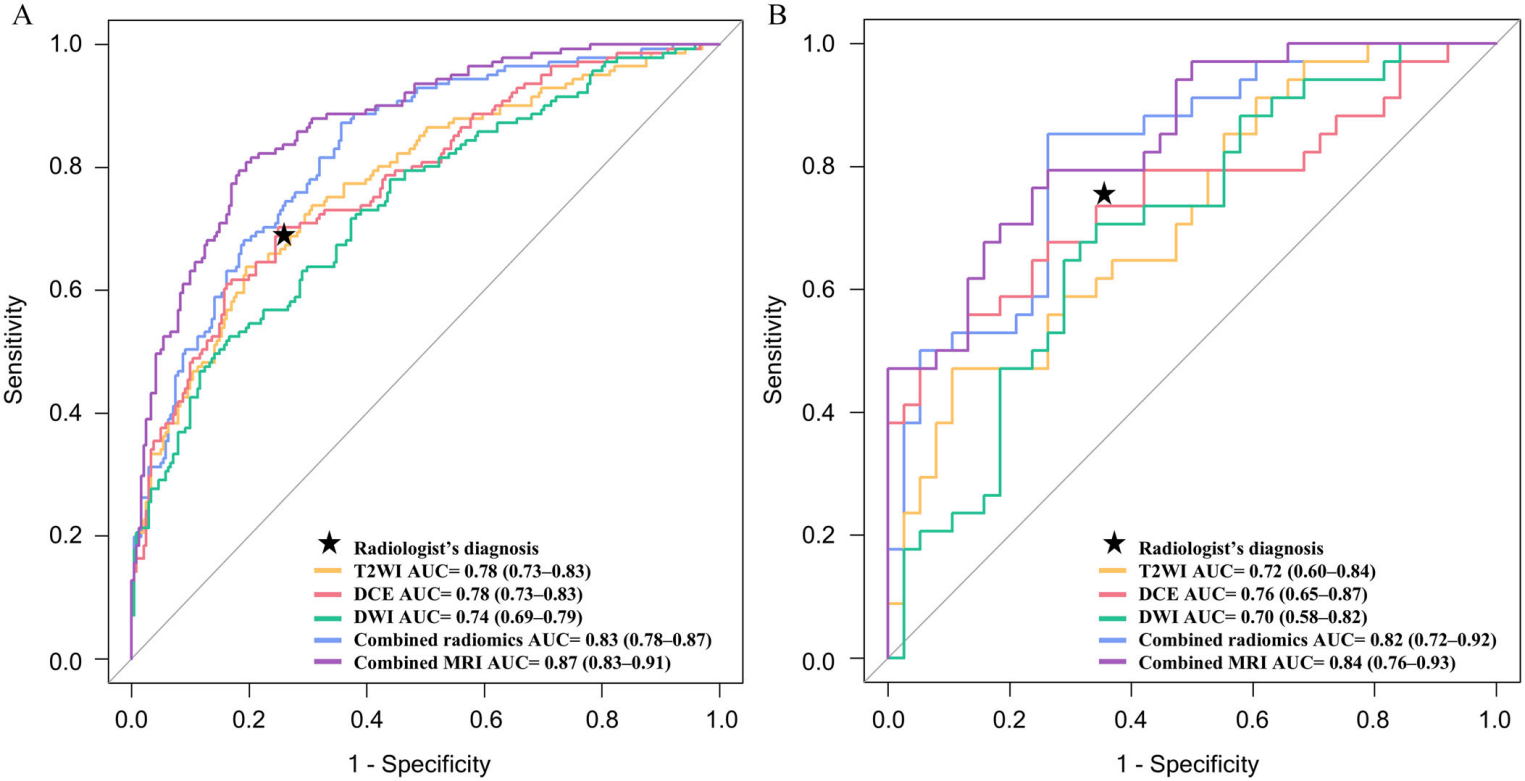
ROC curves and AUC values of different MRI sequence‐based models and radiologist diagnosis performance in the training cohort (A) and the external test cohort (B). AUC, area under curve; Combined MRI, model constructed by integrating the combined radiomics signatures and significant conventional MRI features; combined radiomics, model constructed by integrating radiomics signatures derived from the three MRI sequences; DCE, radiomics model constructed using the DCE‐MRI sequence; DCE, dynamic contrast‐enhancement; DWI, radiomics model constructed using DWI; DWI, diffusion‐weighted imaging; MRI, magnetic resonance imaging; ROC, receiver operating characteristic; T2WI, radiomics model constructed using T2WI; T2WI, T2‐weighted imaging.

Multivariate logistic regression identified multifocality (OR = 3.54, 95% CI: 1.79–7.03), MRI‐reported ALN status (OR = 3.96, 95% CI: 2.29–6.86), and radscore (OR = 5.91, 95% CI: 3.60–9.72) as independent predictors, which formed the final combined MRI model (Table 3). This model achieved an AUC of 0.87 (95% CI: 0.83–0.91) in the training cohort and 0.84 (95% CI: 0.76–0.93) in the external test cohort. While the combined MRI model outperformed the combined radiomics model in the training cohort (*p* < 0.05), it showed no significant difference in performance in the external test cohort (*p* > 0.05) (Table 4). The ROC curves for all models are depicted in Figure 3.

**Table 3:** Univariate and multivariate logistic regression analyses of conventional MRI features and combined radscore.

	Univariate analysis			Multivariate analysis		
Variable	Odds ratio	95% CI	*p*‐value	Odds ratio	95% CI	*p*‐value
Tumor size (mm)	1.08	1.06–1.11	**< 0.001**	0.98	0.95–1.01	0.21
Tumor laterality (L vs. R)	0.86	0.57–1.30	0.47			
FGT (fatty/scattered vs. heterogeneous/extremely dense)	0.86	0.57–1.30	0.48			
BPE (minimal/mild vs. moderate/marked)	1.50	0.84–2.70	0.18			
Multifocality (single vs. multiple)	4.68	2.79–7.82	**< 0.001**	3.54	1.79–7.03	**< 0.001**
Tumor shape (round to oval vs. irregular)	1.47	0.88–2.46	0.15			
Tumor margin (circumscribed vs. not circumscribed)	1.81	0.94–3.45	0.07			
Internal enhancement (homo‐ or heterogeneous vs. rim)	2.60	0.96–7.05	0.06			
Peritumoral edema on T2WI (absent vs. present)	1.58	1.04–2.41	**0.03**	1.09	0.63–1.88	0.76
MRI‐reported ALN status (normal vs. abnormal)	5.96	3.78–9.40	**< 0.001**	3.96	2.29–6.86	**< 0.001**
Radscore	5.49	3.79–7.96	**< 0.001**	5.91	3.60–9.72	**< 0.001**

*Note:* Radscore, the combined radscore for the T2WI+DWI+DCE.

Abbreviations: ALN, axillary lymph node; CI, confidence interval; DCE, dynamic contrast‐enhancement; DWI, diffusion‐weighted imaging; T2WI, T2‐weighted imaging.

**Table 4:** The performance of combined models and radiologist's diagnosis for predicting ALNM in the two cohorts.

Model	AUC (95% CI)	SEN	SPE	ACC	*p*‐value^a^	*p*‐value^b^
Training cohort						
Combined radiomics	0.83 (0.78–0.87)	0.87	0.64	0.73	< 0.001	< 0.001
Combined MRI	0.87 (0.83–0.91)	0.81	0.81	0.81	—	< 0.001
Radiologist's diagnosis	0.71 (0.66–0.76)	0.67	0.74	0.72	—	—
External test cohort						
Combined radiomics	0.82 (0.72–0.92)	0.85	0.74	0.79	0.43	0.09
Combined MRI	0.84 (0.76–0.93)	0.79	0.74	0.76	—	0.001
Radiologist's diagnosis	0.70 (0.60–0.80)	0.77	0.63	0.69	—	—

*Note:* Combined radiomics, the model integrating T2WI, DWI, and DCE; Combined MRI, the model integrating conventional MRI and the combined radiomics; Radiologist's diagnosis, a radiologist assessed the ALN using only MRI.

Abbreviations: ACC, accuracy; AUC, area under curve; CI, confidence interval; DCE, dynamic contrast‐enhancement; DWI, diffusion‐weighted imaging; MRI, magnetic resonance imaging; SEN, sensitivity; SPE, specificity; T2WI, T2‐weighted imaging.

^a^ Comparison of AUC between the combined radiomics model and combined MRI model.

^b^ Comparison of AUC between the radiologist's diagnosis and other models.

### 3.4 Comparison With the Radiologist's Diagnosis

Table 4 shows the performance and comparison of the combined radiomics model, the combined MRI model, and the radiologist's diagnosis. In the training cohort, both the combined MRI and combined radiomics models significantly outperformed the radiologist's diagnosis (*p* < 0.05). In the external test cohort, the combined MRI model significantly outperformed the radiologist's diagnosis (*p* < 0.05). Additionally, in the external test cohort, 30.6% of patients were incorrectly evaluated by the radiologist for ALNM status; the error rate dropped to 23.6% when using the combined MRI model.

### 3.5 Evaluation and Application of the Nomogram

**Figure 4:**
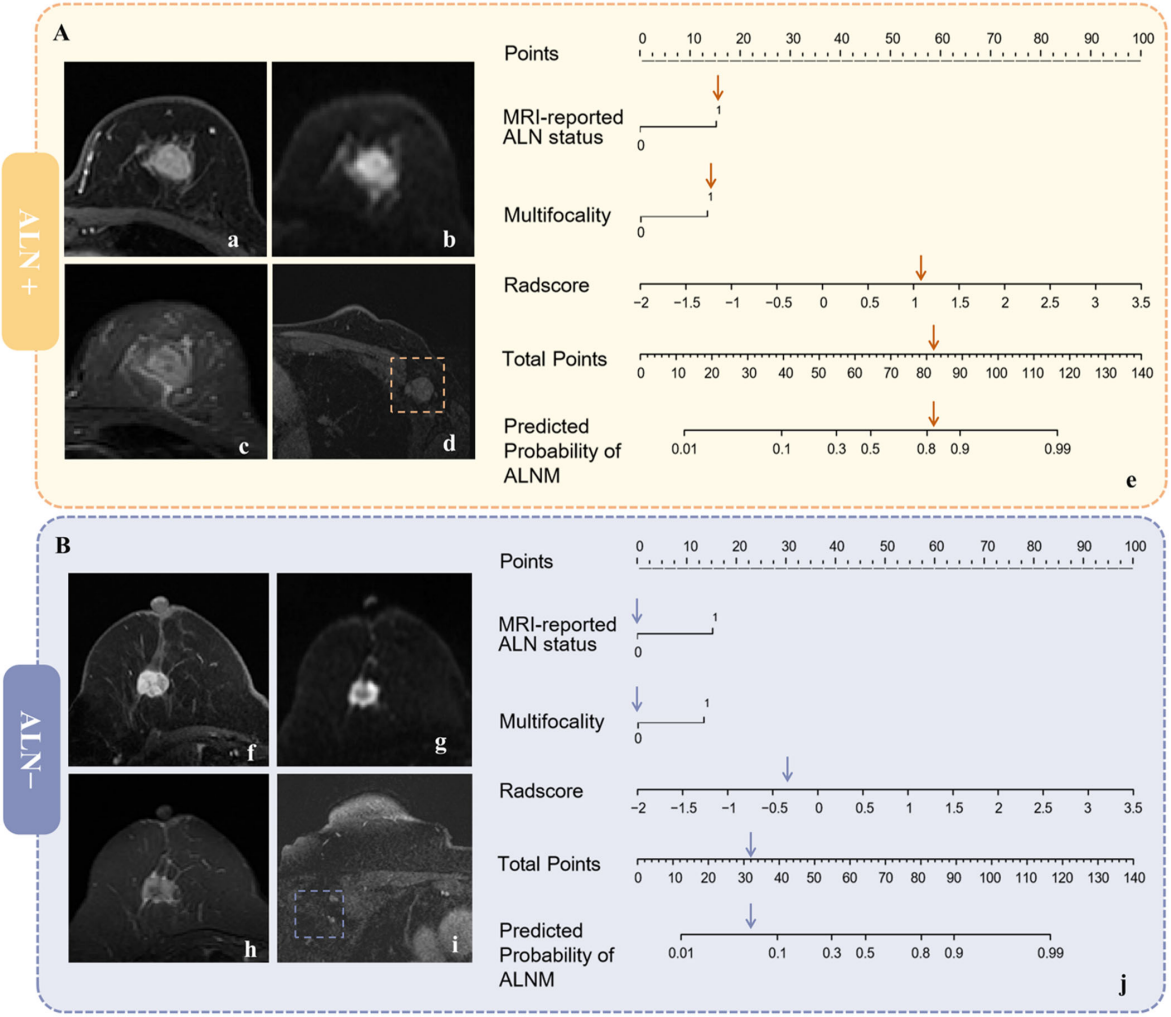
Representative examples of pALN positive and pALN negative. (A) Breast MRI examination of a 60‐year‐old patient with invasive breast cancer, including (a) DCE sequence, (b) DWI sequence, and (c) T2WI sequence. (d) The abnormal ALN reported by MRI was demonstrated on the axial DCE images. (e) The possibility of ALN positivity, assessed by nomogram, could be more than 80%. (B) Breast MRI examination of a 65‐year‐old patient with invasive breast cancer, including (f) DCE sequence, (g) DWI sequence, and (h) T2WI sequence. (i) The normal ALN reported by MRI was demonstrated on the axial DCE images. (j) The possibility of ALN positive assessed by the nomogram could be less than 10%. ALN+, axillary lymph node positive; ALN−, axillary lymph node negative; DCE, dynamic contrast‐enhancement; DWI, diffusion‐weighted imaging; MRI, magnetic resonance imaging; T2WI, T2‐weighted imaging.

A nomogram based on the combined MRI model for predicting ALNM is displayed in Supporting Information: Figure S1. The accuracy of this nomogram was evaluated using a calibration curve, which confirmed the alignment between actual and predicted ALNM risks (Supporting Information: Figure S2). DCA demonstrated that the nomogram provided substantial net benefits across various high‐risk thresholds (Supporting Information: Figure S3). Representative examples of the nomogram's application for ALNM status prediction are shown in Figure 4.

### 3.6 Survival Analysis

**Figure 5:**
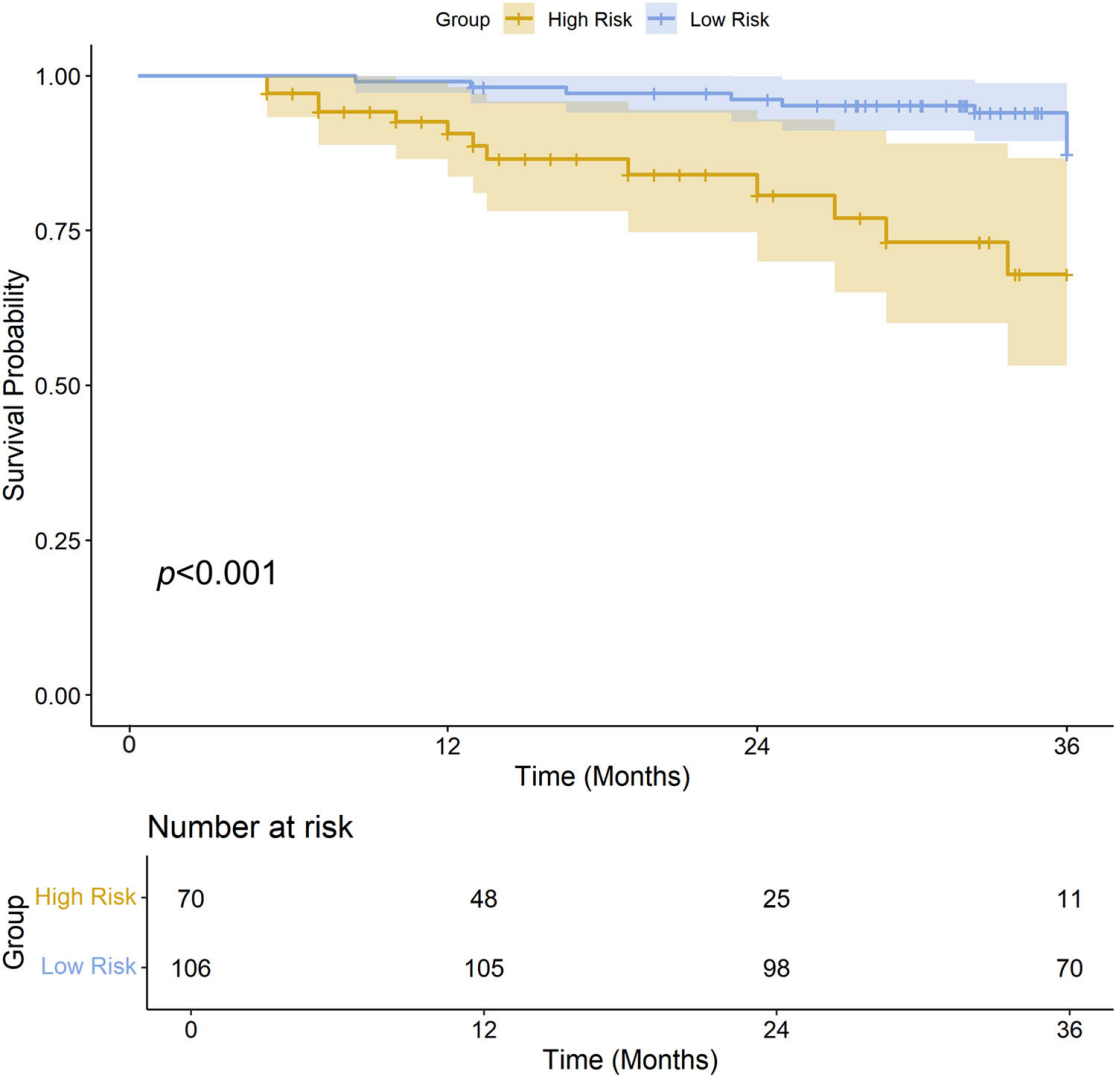
KM curve of 3 years DFS stratified by risk groups based on the combined MRI model. The high‐risk group (yellow) exhibited significantly shorter DFS compared to the low‐risk group (blue), with a *p* < 0.001. Shaded regions represent the 95% confidence intervals. The “Number at risk” table below the graph indicates the number of patients remaining at risk at various time points for each group. DFS, disease‐free survival; KM, Kaplan–Meier.

A total of 176 patients were included in the survival analysis; the median follow‐up was 33.9 months (IQR: 19.3–36.0 months). During the follow‐up period, 24 patients (13.6%) experienced recurrence. Patients were classified into low‐risk and high‐risk groups on the basis of the model's predictions with a cutoff value of 0.42. The KM curve demonstrated that a high‐risk score was significantly associated with shorter DFS (HR = 3.85, 95% CI: 1.68–8.82, *p* < 0.001, Figure 5).

## 4 Discussion

In this study, we developed and evaluated single‐sequence and combined radiomics models to assess ALNM status in invasive breast cancer patients. In the external test cohort, the DCE model achieved the highest AUC among single‐sequence models, although the differences compared with T2WI and DWI were not statistically significant. The combined radiomics model outperformed the single‐sequence models in AUC, sensitivity, and accuracy, although it did not achieve a significantly higher AUC compared with the DCE model. Additionally, the combined MRI model, which incorporated conventional MRI features, slightly surpassed the combined radiomics model in AUC and significantly outperformed the radiologist's diagnosis. Survival analysis demonstrated the clinical value of the combined MRI model in predicting prognosis based on the ALNM risk score.

Previous research has used DCE, DWI, and T2WI to predict ALNM status in breast cancer and generally indicated that DCE and DWI are equally effective and both surpass T2WI in performance [18]. These findings partially diverge from those observed in our investigation. In our analysis, DCE was comparable to T2WI and outperformed DWI in terms of efficacy within the training cohort. In the external test cohort, although DCE registered the highest AUC, the distinctions among DCE, T2WI, and DWI did not reach statistical significance. Nonetheless, the trend across both cohorts suggests DCE as potentially the most robust single‐sequence model, consistently demonstrating superior AUCs. The superior performance of DCE may be linked to its ability to capture key characteristics related to tumor vascular invasion and angiogenesis, as highlighted in previous research [17, 30]. Additionally, the DCE images were captured during the second post‐contrast phase, which is known for providing clear contrast between the tumor and adjacent tissues [25, 26]. This phase likely enhances the visualization of tumor microvasculature and vascular permeability, factors integral to assessing tumor aggressiveness and metastatic potential. In contrast, T2WI, while sensitive to water content and effective in identifying edema [31], was less effective in this study, likely as a result of the omission of peritumoral feature extraction. Similarly, although DWI reflects water motion and tumor microenvironment heterogeneity [32], it may not be as effective for predicting ALNM status, as it lacks the sensitivity to vascular factors like tumor microvascular infiltration and angiogenesis, which are better depicted by DCE [17].

Prior research has suggested that multi‐sequence models can provide additional insights and improve prediction accuracy compared with single‐sequence models [19]. However, another study showed that multi‐sequence MRI does not significantly enhance the predictive efficacy for ALNM status compared with single‐sequence models [18]. In our study, the combined radiomics model displayed superior AUC values compared with the single‐sequence models, notably surpassing those of DWI and T2WI. Although the difference in AUC between the combined model and DCE did not reach statistical significance, the accuracy of the combined model proved to be greater. This suggests that synthesizing information from various sequences may yield more holistic insights and improve the ability to predict ALN status.

In the univariate analysis of conventional MRI features, a significant association was observed between larger tumor size, multifocality, peritumoral edema, MRI‐reported ALN abnormalities, and pALN positivity. Larger tumor sizes and multifocal lesions have been linked to a higher tumor burden, consequently elevating the likelihood of ALNM [33, 34]. MRI‐reported ALN status also emerged as an independent predictor of ALNM, aligning with findings from previous studies [33, 35]. Furthermore, peritumoral edema is linked to enhanced tumor invasiveness and indicative of microvascular infiltration [36], supporting the notion that tumor spread and metastasis are driven by peritumoral infiltration [37].

The combined MRI model, constructed by integrating conventional MRI features with the optimal radiomics model, exhibited robust performance in both the training and external test cohorts. This result indicated better performance compared with results from a previous study that used MRI quantitative features and clinicopathological factors to predict ALNM status, which reported an AUC of 0.799 [9]. This highlights the ability of multiparametric MRI‐based radiomics features to reveal deeper tumor characteristics, enabling more effective identification of ALNM‐associated biomarkers and superior predictive performance. Moreover, compared with the radiologist's diagnosis, the combined MRI model significantly improved ALNM prediction accuracy and enhanced diagnostic precision. To facilitate clinical decision‐making, we developed a nomogram based on this model, offering an interpretable and intuitive tool for practical application.

To further assess the clinical utility, we compared the DFS of patients stratified into high‐ and low‐risk groups on the basis of the model's predictions. Survival analysis showed a significant difference in DFS between the two groups, with a higher ALNM risk associated with shorter DFS. This suggests that the combined MRI model not only predicts ALNM but also provides valuable prognostic information, indicating its potential to guide clinical decision‐making and patient management.

This study has several limitations. First, its retrospective nature and the relatively small size of the external test cohort may limit the generalizability of the findings. Expanding the sample size and including multicenter data may enhance the robustness and applicability of the model's predictive performance. Second, the study relied on manual delineation of ROIs, a process that is both time‐intensive and potentially inefficient. Further methodological optimization to improve efficiency is required. Additionally, the study did not include peritumoral radiomic features, which are known to reflect the tumor microenvironment and are associated with tumor growth and invasiveness [38]. Incorporating peritumoral features may yield more comprehensive insights into predicting ALNM status in breast cancer patients.

## 5 Conclusion

The combined radiomics model outperformed the single‐sequence models in predicting ALNM status. The combined MRI model achieved the highest predictive performance and showed significantly improved diagnostic accuracy compared with radiologists. Moreover, risk stratification based on this model revealed significant prognostic differences in patients with different ALNM status, demonstrating its potential clinical value.

## Author Contributions

**Qingcong Kong:** conceptualization (equal), formal analysis (equal), funding acquisition (equal), writing – review and editing (equal). **Yongxin Chen:** data curation (equal), investigation (equal), methodology (equal), writing – original draft (equal). **Yi Sui:** data curation (equal), formal analysis (equal), writing – original draft (equal). **Siyi Chen:** methodology (equal), visualization (equal). **Xinghan Lv:** data curation (equal), validation (equal). **Wenjie Tang:** conceptualization (equal), funding acquisition (equal). **Zhidan Zhong:** formal analysis (equal), investigation (equal). **Xiaomeng Yu:** data curation (equal), visualization (equal). **Kuiming Jiang:** project administration (equal), validation (equal). **Lei Zhang:** methodology (equal), supervision (equal). **Jianning Chen:** conceptualization (equal), supervision (equal). **Jie Qin:** conceptualization (equal), supervision (equal), writing – review and editing (equal). **Yuan Guo:** conceptualization (equal), funding acquisition (equal), project administration (equal), writing – review and editing (equal).

## Ethics Statement

All procedures performed in studies involving human participants were in accordance with the ethical standards of the institutional and/or national research committee and with the 1964 Helsinki Declaration and its later amendments or comparable ethical standards. This retrospective study was approved by the Ethics Committee Review Board of Guangzhou First People's Hospital (Approval Number: S202308301) and the Third Affiliated Hospital of Sun Yat‐Sen University (Approval Number: SL‐RG2024‐070‐01).

## Consent

Due to the nature of the retrospective study, the requirement for informed consent was waived.

## Conflicts of Interest

The authors declare no conflicts of interest.

## Supporting information

supplementary materials‐final.

## Data Availability

Data that support the findings of this study are available from the corresponding author upon reasonable request.

## References

[1] L. Li , Y. Wu , B. Lan , and F. Ma , “Efficacy and Safety of First‐Line Regimens for Advanced HER2‐Positive Breast Cancer: A Bayesian Network Meta‐Analysis,” Cancer Innovation 3, no. 4 (2024): e126, 10.1002/cai2.126. 38948247 PMC11212280

[2] Z. Wang , L. Yu , X. Ding , X. Liao , and L. Wang , “Lymph Node Metastasis Prediction From Whole Slide Images With Transformer‐Guided Multiinstance Learning and Knowledge Transfer,” IEEE Transactions on Medical Imaging 41, no. 10 (2022): 2777–2787, 10.1109/TMI.2022.3171418. 35486559

[3] J. Gao , X. Zhong , W. Li , et al., “Attention‐Based Deep Learning for the Preoperative Differentiation of Axillary Lymph Node Metastasis in Breast Cancer on DCE‐MRI,” Journal of Magnetic Resonance Imaging 57, no. 6 (2023): 1842–1853, 10.1002/jmri.28464. 36219519

[4] F. Maxwell , C. de Margerie Mellon , M. Bricout , et al., “Diagnostic Strategy for the Assessment of Axillary Lymph Node Status in Breast Cancer,” Diagnostic and Interventional Imaging 96, no. 10 (2015): 1089–1101, 10.1016/j.diii.2015.07.007. 26372221

[5] M. Chen , C. Kong , G. Lin , et al., “Development and Validation of Convolutional Neural Network‐Based Model to Predict the Risk of Sentinel or Non‐Sentinel Lymph Node Metastasis in Patients With Breast Cancer: A Machine Learning Study,” EClinicalMedicine 63 (2023): 102176, 10.1016/j.eclinm.2023.102176. 37662514 PMC10474371

[6] A. E. Giuliano , K. K. Hunt , K. V. Ballman , et al., “Axillary Dissection vs No Axillary Dissection in Women With Invasive Breast Cancer and Sentinel Node Metastasis: A Randomized Clinical Trial,” Journal of the American Medical Association 305, no. 6 (2011): 569–575, 10.1001/jama.2011.90. 21304082 PMC5389857

[7] H. Tan , F. Gan , Y. Wu , et al., “Preoperative Prediction of Axillary Lymph Node Metastasis in Breast Carcinoma Using Radiomics Features Based on the Fat‐Suppressed T2 Sequence,” Academic Radiology 27, no. 9 (2020): 1217–1225, 10.1016/j.acra.2019.11.004. 31879160

[8] M. A. Marino , D. Avendano , P. Zapata , C. C. Riedl , and K. Pinker , “Lymph Node Imaging in Patients With Primary Breast Cancer: Concurrent Diagnostic Tools,” Oncologist 25, no. 2 (2020): e231–e242, 10.1634/theoncologist.2019-0427. 32043792 PMC7011661

[9] X. Chen , Z. Yang , R. Huang , et al., “Development and Validation of a Point‐Based Scoring System for Predicting Axillary Lymph Node Metastasis and Disease Outcome in Breast Cancer Using Clinicopathological and Multiparametric MRI Features,” Cancer Imaging 23, no. 1 (2023): 54, 10.1186/s40644-023-00564-9. 37264446 PMC10233973

[10] Z. Li , Y. Gao , H. Gong , et al., “Different Imaging Modalities for the Diagnosis of Axillary Lymph Node Metastases in Breast Cancer: A Systematic Review and Network Meta‐Analysis of Diagnostic Test Accuracy,” Journal of Magnetic Resonance Imaging 57, no. 5 (2023): 1392–1403, 10.1002/jmri.28399. 36054564

[11] R. J. Gillies , P. E. Kinahan , and H. Hricak , “Radiomics: Images Are More Than Pictures, They Are Data,” Radiology 278, no. 2 (2016): 563–577, 10.1148/radiol.2015151169. 26579733 PMC4734157

[12] Y. Zhu , L. Yang , and H. Shen , “Value of the Application of CE‐MRI Radiomics and Machine Learning in Preoperative Prediction of Sentinel Lymph Node Metastasis in Breast Cancer,” Frontiers in Oncology 11 (2021): 757111, 10.3389/fonc.2021.757111. 34868967 PMC8640128

[13] A. S. Tagliafico , M. Piana , D. Schenone , R. Lai , A. M. Massone , and N. Houssami , “Overview of Radiomics in Breast Cancer Diagnosis and Prognostication,” Breast 49 (2020): 74–80, 10.1016/j.breast.2019.10.018. 31739125 PMC7375670

[14] Y. Yu , Y. Tan , C. Xie , et al., “Development and Validation of a Preoperative Magnetic Resonance Imaging Radiomics‐Based Signature to Predict Axillary Lymph Node Metastasis and Disease‐Free Survival in Patients With Early‐Stage Breast Cancer,” JAMA Network Open 3, no. 12 (2020): e2028086, 10.1001/jamanetworkopen.2020.28086. 33289845 PMC7724560

[15] O. Lafcı , P. Celepli , P. Seher Öztekin , and P. N. Koşar , “DCE‐MRI Radiomics Analysis in Differentiating Luminal A and Luminal B Breast Cancer Molecular Subtypes,” Academic Radiology 30, no. 1 (2023): 22–29, 10.1016/j.acra.2022.04.004. 35595629

[16] M. Liu , N. Mao , H. Ma , et al., “Pharmacokinetic Parameters and Radiomics Model Based on Dynamic Contrast Enhanced MRI for the Preoperative Prediction of Sentinel Lymph Node Metastasis in Breast Cancer,” Cancer Imaging 20, no. 1 (2020): 65, 10.1186/s40644-020-00342-x. 32933585 PMC7493182

[17] D. Song , F. Yang , Y. Zhang , et al., “Dynamic Contrast‐Enhanced MRI Radiomics Nomogram for Predicting Axillary Lymph Node Metastasis in Breast Cancer,” Cancer Imaging 22, no. 1 (2022): 17, 10.1186/s40644-022-00450-w. 35379339 PMC8981871

[18] R. Chai , H. Ma , M. Xu , et al., “Differentiating Axillary Lymph Node Metastasis in Invasive Breast Cancer Patients: A Comparison of Radiomic Signatures From Multiparametric Breast MR Sequences,” Journal of Magnetic Resonance Imaging 50, no. 4 (2019): 1125–1132, 10.1002/jmri.26701. 30848041 PMC8579490

[19] Z. Wang , H. Sun , J. Li , et al., “Preoperative Prediction of Axillary Lymph Node Metastasis in Breast Cancer Using CNN Based on Multiparametric MRI,” Journal of Magnetic Resonance Imaging 56, no. 3 (2022): 700–709, 10.1002/jmri.28082. 35108415

[20] A. E. Giuliano , J. L. Connolly , S. B. Edge , et al., “Breast Cancer—Major Changes in the American Joint Committee on Cancer Eighth Edition Cancer Staging Manual,” CA: A Cancer Journal for Clinicians 67, no. 4 (2017): 290–303, 10.3322/caac.21393. 28294295

[21] E. A. Rakha and A. R. Green , “Molecular Classification of Breast Cancer: What the Pathologist Needs to Know,” Pathology 49, no. 2 (2017): 111–119, 10.1016/j.pathol.2016.10.012. 28040199

[22] M. Ma , R. Liu , C. Wen , et al., “Predicting the Molecular Subtype of Breast Cancer and Identifying Interpretable Imaging Features Using Machine Learning Algorithms,” European Radiology 32, no. 3 (2022): 1652–1662, 10.1007/s00330-021-08271-4. 34647174

[23] Q. Wang , Y. Lin , C. Ding , et al., “Multi‐Modality Radiomics Model Predicts Axillary Lymph Node Metastasis of Breast Cancer Using MRI and Mammography,” European Radiology 34, no. 9 (2024): 6121–6131, 10.1007/s00330-024-10638-2. 38337068

[24] N. Mao , Y. Dai , F. Lin , et al., “Radiomics Nomogram of DCE‐MRI for the Prediction of Axillary Lymph Node Metastasis in Breast Cancer,” Frontiers in Oncology 10 (2020): 541849, 10.3389/fonc.2020.541849. 33381444 PMC7769044

[25] W. Tang , Q. Kong , Z. Cheng , et al., “Performance of Radiomics Models for Tumour‐Infiltrating Lymphocyte (TIL) Prediction in Breast Cancer: The Role of the Dynamic Contrast‐Enhanced (DCE) MRI Phase,” European Radiology 32, no. 2 (2022): 864–875, 10.1007/s00330-021-08173-5. 34430998

[26] S. Peng , L. Chen , J. Tao , et al., “Radiomics Analysis of Multi‐Phase DCE‐MRI in Predicting Tumor Response to Neoadjuvant Therapy in Breast Cancer,” Diagnostics 11, no. 11 (2021): 2086, 10.3390/diagnostics11112086. 34829433 PMC8625316

[27] L. Prieto , R. Lamarca , A. Casado , and J. Alonso , “The Evaluation of Agreement on Continuous Variables by the Intraclass Correlation Coefficient,” Journal of Epidemiology & Community Health 51, no. 5 (1997): 579–581, 10.1136/jech.51.5.579-a. PMC1060549 9425473

[28] Z. Xu , Y. Xie , L. Wu , et al., “Using Machine Learning Methods to Assess Lymphovascular Invasion and Survival in Breast Cancer: Performance of Combining Preoperative Clinical and MRI Characteristics,” Journal of Magnetic Resonance Imaging 58, no. 5 (2023): 1580–1589, 10.1002/jmri.28647. 36797654

[29] J. R. Landis and G. G. Koch , “The Measurement of Observer Agreement for Categorical Data,” Biometrics 33, no. 1 (1977): 159–174. 843571

[30] L. Han , Y. Zhu , Z. Liu , et al., “Radiomic Nomogram for Prediction of Axillary Lymph Node Metastasis in Breast Cancer,” European Radiology 29, no. 7 (2019): 3820–3829, 10.1007/s00330-018-5981-2. 30701328

[31] Y. Chen , L. Wang , R. Luo , H. Liu , Y. Zhang , and D. Wang , “Focal Breast Edema and Breast Edema Score on T2‐Weighted Images Provides Valuable Biological Information for Invasive Breast Cancer,” Insights Into Imaging 14, no. 1 (2023): 73, 10.1186/s13244-023-01424-7. 37121926 PMC10149534

[32] F. Galati , V. Rizzo , R. M. Trimboli , E. Kripa , R. Maroncelli , and F. Pediconi , “MRI as a Biomarker for Breast Cancer Diagnosis and Prognosis,” BJR Open 4, no. 1 (2022): 20220002, 10.1259/bjro.20220002. 36105423 PMC9459861

[33] Y. Chen , J. Li , J. Zhang , Z. Yu , and H. Jiang , “Radiomic Nomogram for Predicting Axillary Lymph Node Metastasis in Patients With Breast Cancer,” Academic Radiology 31, no. 3 (2024): 788–799, 10.1016/j.acra.2023.10.026. 37932165

[34] W. Zhang , S. Wang , Y. Wang , et al., “Ultrasound‐Based Radiomics Nomogram for Predicting Axillary Lymph Node Metastasis in Early‐Stage Breast Cancer,” La Radiologia Medica 129, no. 2 (2024): 211–221, 10.1007/s11547-024-01768-0. 38280058

[35] W. Chen , G. Lin , C. Kong , et al., “Non‐Invasive Prediction Model of Axillary Lymph Node Status in Patients With Early‐Stage Breast Cancer: A Feasibility Study Based on Dynamic Contrast‐Enhanced‐MRI Radiomics,” British Journal of Radiology 97, no. 1154 (2024): 439–450, 10.1093/bjr/tqad034. 38308028

[36] J. Zhou , Y. Zhang , H. Miao , et al., “Preoperative Differentiation of HER2‐Zero and HER2‐Low From HER2‐Positive Invasive Ductal Breast Cancers Using BI‐RADS MRI Features and Machine Learning Modeling,” Journal of Magnetic Resonance Imaging 61, no. 2 (2025): 928–941, 10.1002/jmri.29447. 38726477 PMC11550260

[37] H. Zhang , Y. Dong , X. Jia , et al., “Comprehensive Risk System Based on Shear Wave Elastography and BI‐RADS Categories in Assessing Axillary Lymph Node Metastasis of Invasive Breast Cancer—A Multicenter Study,” Frontiers in Oncology 12 (2022): 830910, 10.3389/fonc.2022.830910. 35359391 PMC8960926

[38] J. Zhou , Y. Zhang , K. T. Chang , et al., “Diagnosis of Benign and Malignant Breast Lesions on DCE‐MRI by Using Radiomics and Deep Learning With Consideration of Peritumor Tissue,” Journal of Magnetic Resonance Imaging 51, no. 3 (2020): 798–809, 10.1002/jmri.26981. 31675151 PMC7709823

