# Supplementary material for: The Role of Multiparametric MRI Radiomics for Preoperative Prediction of Axillary Lymph Node Metastasis in Patients With Invasive Breast Cancer: A Comparative Study
Source: Cancer Innov. 2025 Jul 13;4(5):e70022. doi: 10.1002/cai2.70022 (PMC12256143; doi:10.1002/cai2.70022)
Supplement: Supplementary file 1 — supplementary materials‐final. [file CAI2-4-e70022-s001.doc]

**Supplementary materials**

**Table S1. MRI Scan Sequences and Parameters of Two Centers**

| **Scanner, Sequence** | **TR (ms)** | **TE (ms)** | **FOV (mm^2^)** | **Slice Thickness (mm)** | **Acquisition Time (sec)** | **b values（s/mm^2^)** |
| --- | --- | --- | --- | --- | --- | --- |
| Center 1 | | | | | | |
| United Imaging 1.5 T  (uMR 560) |  |  |  |  |  |  |
| T2WI | 3800 | 42.7 | 328×350 | 4.0 | 126 | − |
| DWI | 3800 | 78.4 | 350×200 | 4.0 | 103 | 0, 800 |
| DCE | 5.1 | 2.1 | 320×320 | 2.4 | 394 | − |
| Center 2 | | | | | | |
| GE 1.5 T  (BRIVO MR355) |  |  |  |  |  |  |
| T2WI | 7266 | 42 | 330×330 | 4.0 | 153 | − |
| DWI | 6277 | 57 | 330×330 | 4.0 | 157 | 0, 800 |
| DCE | 5.2 | 2.4 | 350×350 | 3.0 | 429 | − |

TR, repetition time; TE, echo time; FOV, field of view; T2WI, T2-weighted imaging; DWI, diffusion-weighted imaging; DCE, dynamic contrast-enhanced.

**Table S2. The Clinicopathologic Characteristics of Patients in Two Cohorts**

| Characteristics | Training cohort  (*n*=382) | External Test cohort  (*n*=72) | *p* value |
| --- | --- | --- | --- |
| Age(years) (mean ± sd) | 51.2±10.5 | 49.3±11.8 | 0.18 |
| Menstrual status, *n* (%) |  |  | 0.69 |
| Menopause | 248 (64.9) | 45 (62.5) |  |
| Menses | 134 (35.1) | 27 (37.5) |  |
| ER, *n* (%) |  |  | 0.16 |
| Positive | 285 (74.6) | 48 (66.7) |  |
| Negative | 97 (25.4) | 24 (33.3) |  |
| PR, *n* (%) |  |  | 0.12 |
| Positive | 264 (69.1) | 43 (59.7) |  |
| Negative | 118 (30.9) | 29 (40.3) |  |
| HER2, *n* (%) |  |  | 0.06 |
| Positive | 111 (29.1) | 29 (40.3) |  |
| Negative | 271 (70.9) | 43 (59.7) |  |
| Ki-67, *n* (%) |  |  | 0.16 |
| Low | 87 (22.8) | 11 (15.3) |  |
| High | 295 (77.2) | 61 (84.7) |  |
| pALN, *n* (%) |  |  | 0.10 |
| Positive | 141 (36.9) | 34 (47.2) |  |
| Negative | 241 (63.1) | 38 (52.8) |  |

ER, estrogen receptor; PR, progesterone receptor; HER2, human epidermal growth factor receptor-2; pALN, pathologic axillary lymph node.

**Table S3.** Interreader agreement for conventional MRI features

| Feature | Agreement (95% CI)^a^ |
| --- | --- |
| Tumor size | 0.94 (0.86–0.98) |
| Fibroglandular tissue | 0.85 (0.80–0.91) |
| Background parenchymal enhancement | 0.83 (0.75–0.91) |
| Multifocality | 0.91 (0.86–0.96) |
| Tumor shape | 0.78 (0.72–0.85) |
| Tumor margin | 0.81 (0.75–0.87) |
| Mass internal enhancement | 0.80 (0.68–0.91) |
| Peritumoral edema on T2WI | 0.86 (0.80–0.91) |
| MRI-reported ALN status | 0.81 (0.75–0.87) |

^a^Expressed as intraclass correlation coefficient for tumor size and as kappa coefficient for remaining variables.

ALN = axillary lymph nodes

Table S4. The Selected Radiomics Features of Three Single-sequence Models

| **Model** | **Features** | **Coefficient** |
| --- | --- | --- |
| **DCE (12)** | original_shape_Sphericity | −0.28081703 |
|  | original_glcm_InverseVariance | 0.046545358 |
|  | wavelet-LHL_glcm_Imc2 | −0.071445743 |
|  | wavelet-HLL_firstorder_Skewness | 0.001282897 |
|  | wavelet-HLH_gldm_DependenceVariance | −0.031000174 |
|  | wavelet-HLH_gldm_SmallDependenceLowGrayLevelEmphasis | −0.208952083 |
|  | wavelet-HHL_gldm_DependenceVariance | 0.170763866 |
|  | log-sigma-3-0-mm-3D_glszm_SizeZoneNonUniformity | 0.175152009 |
|  | log-sigma-3-0-mm-3D_ngtdm_Busyness | 0.117749427 |
|  | log-sigma-5-0-mm-3D_firstorder_Maximum | 0.035836915 |
|  | log-sigma-5-0-mm-3D_gldm_DependenceNonUniformityNormalized | −0.086088877 |
|  | log-sigma-5-0-mm-3D_ngtdm_Contrast | −0.09550573 |
| **DWI (3)** | original_shape_Sphericity | −0.181915428 |
|  | wavelet-LHL_glcm_Idn | 0.327024635 |
|  | log-sigma-5-0-mm-3D_glcm_Idn | 0.038534125 |
| **T2WI (15)** | original_shape_Sphericity | −0.342812992 |
|  | wavelet-LLH_glszm_GrayLevelNonUniformityNormalized | 0.039658149 |
|  | wavelet-LLH_gldm_DependenceEntropy | −0.116403077 |
|  | wavelet-LLH_ngtdm_Busyness | 0.05154934 |
|  | wavelet-LHL_ngtdm_Busyness | 0.002955234 |
|  | wavelet-LHH_firstorder_Skewness | −0.085736371 |
|  | wavelet-HLH_ngtdm_Busyness | 0.294457299 |
|  | wavelet-HHL_firstorder_Mean | 0.072442218 |
|  | wavelet-HHH_glszm_ZoneEntropy | 0.013679066 |
|  | log-sigma-1-0-mm-3D_ngtdm_Contrast | −0.234981691 |
|  | log-sigma-3-0-mm-3D_firstorder_Kurtosis | 0.02985378 |
|  | log-sigma-3-0-mm-3D_glcm_Idn | 0.11323455 |
|  | log-sigma-3-0-mm-3D_gldm_DependenceVariance | 0.056992402 |
|  | log-sigma-5-0-mm-3D_firstorder_Skewness | −0.031839873 |
|  | log-sigma-5-0-mm-3D_glcm_Imc1 | −0.131668768 |

**Table S5. The Selected Radiomics Features of Combined Radiomics Model**

| **Sequence** | **Features** | **Coefficient** |
| --- | --- | --- |
| **DCE (9)** | original_glcm_InverseVariance | 0.036430417 |
|  | wavelet-HLH_gldm_SmallDependenceLowGrayLevelEmphasis | −0.171796138 |
|  | wavelet-HHL_gldm_DependenceVariance | 0.197857644 |
|  | log-sigma-3-0-mm-3D_glszm_SizeZoneNonUniformity | 0.028413156 |
|  | log-sigma-3-0-mm-3D_ngtdm_Busyness | 0.028076653 |
|  | log-sigma-5-0-mm-3D_firstorder_Kurtosis | 0.018306093 |
|  | log-sigma-5-0-mm-3D_firstorder_Maximum | 0.041296571 |
|  | log-sigma-5-0-mm-3D_gldm_DependenceNonUniformityNormalized | −0.083903068 |
|  | log-sigma-5-0-mm-3D_ngtdm_Contrast | −0.01054662 |
| **DWI (5)** | original_shape_Sphericity | −0.217596161 |
|  | waveletLLH_firstorder_Skewness | −0.036623219 |
|  | waveletLHL_glcm_Idn | 0.292338727 |
|  | waveletLHH_glszm_ZoneEntropy | 0.008656048 |
|  | waveletHHL_glszm_GrayLevelNonUniformityNormalized | 0.091971674 |
| **T2WI (10)** | original_shape_Sphericity | −0.247081596 |
|  | waveletLLH_glcm_Imc1 | 0.023377345 |
|  | waveletLLH_glszm_GrayLevelNonUniformityNormalized | 0.002940429 |
|  | waveletLLH_gldm_DependenceEntropy | −0.199820338 |
|  | waveletLLH_ngtdm_Busyness | 0.011404794 |
|  | waveletLHH_firstorder_Skewness | −0.05820358 |
|  | waveletHHL_firstorder_Mean | 0.04737164 |
|  | logsigma30mm3D_gldm_DependenceVariance | 0.004962077 |
|  | logsigma30mm3D_ngtdm_Contrast | −0.031931617 |
|  | logsigma50mm3D_glcm_Imc1 | −0.01940109 |


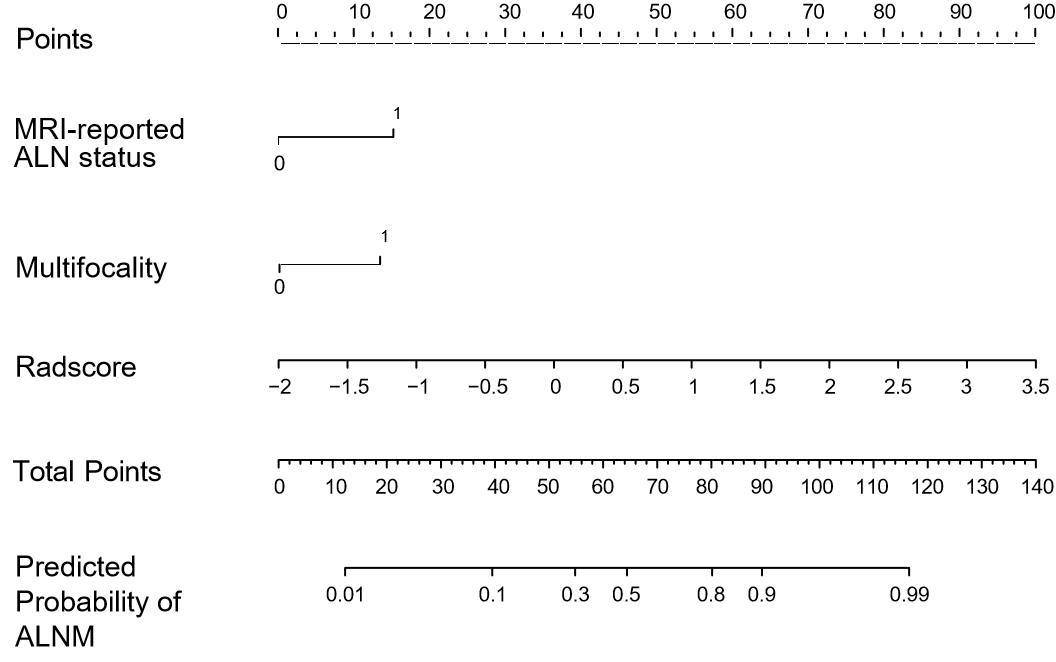


**Figure S1.** A nomogram for predicting ALNM in patients with invasive breast cancer. The nomogram was composed of multifocality, MRI-reported ALN status and Radscore. MRI, magnetic resonance imaging; ALN, axillary lymph node; Radscore, radscore of the model integrating the radiomics signature based on T2WI, DWI and DCE; ALNM, axillary lymph node metastasis.


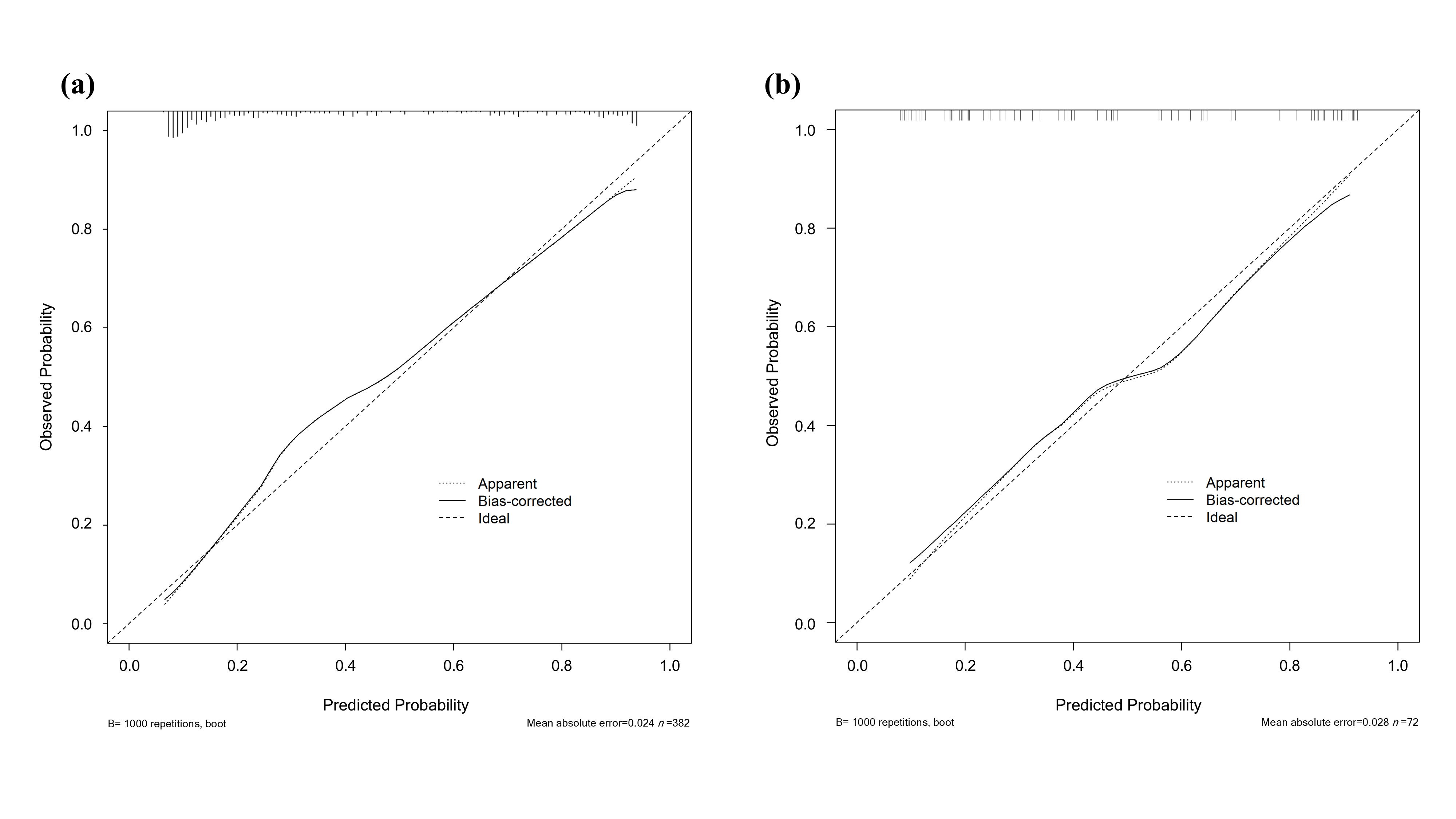


**Figure S2.** Calibration curves of nomogram in the training **(a)** and external test **(b)** cohorts. The curves showed a good calibration of the model in two cohorts.


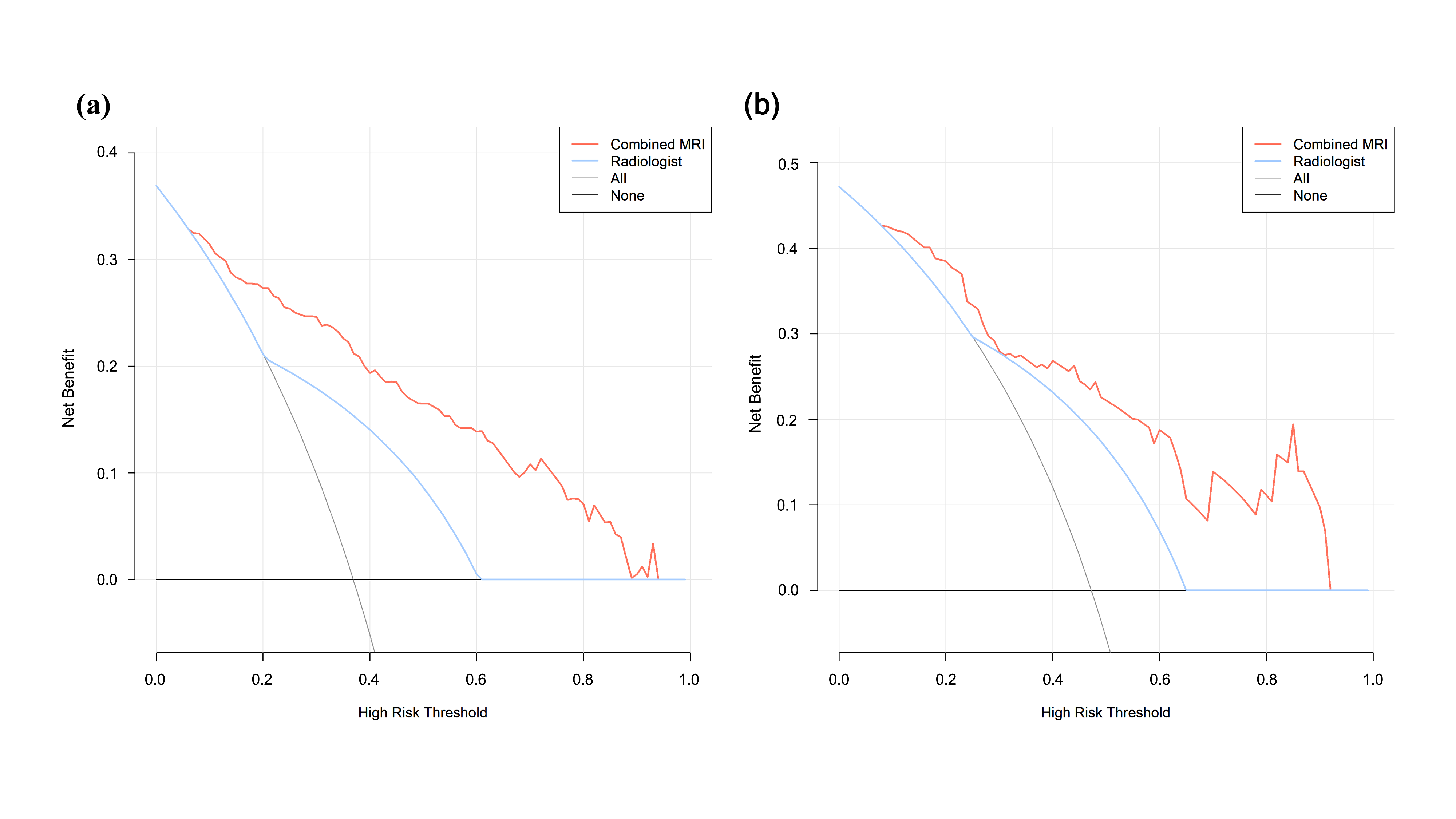


**Figure S3.** DCA for Combined MRI model and the radiologist’s diagnosis in the training **(a)** and external test **(b)** cohorts; the y-axis indicates the net benefit, the x-axis indicates threshold probability.
